# Validation of the ATN Biomarker Framework for Alzheimer's Disease in Latin American Population

**DOI:** 10.1002/alz70856_103030

**Published:** 2025-12-25

**Authors:** Claudia Duran‐Aniotz

**Affiliations:** ^1^ Latin American Brain Health Institute (BrainLat), Universidad Adolfo Ibañez, Santiago, Chile

## Abstract

Plasma biomarkers hold promise for improving dementia diagnosis but remain insufficiently validated in diverse populations. While AT(N) biomarkers for Alzheimer's disease (AD) and frontotemporal lobar degeneration (FTLD) are widely used in high‐income countries, their evaluation in Latin America (LA) is limited, despite the region's genetic, lifestyle, and diagnostic complexities. This study addresses these gaps by evaluating plasma biomarkers (Aβ40, Aβ42, Aβ42/Aβ40 ratio, *p*‐tau181, and NfL) in a multicountry cohort spanning five LA countries (*n* = 637). Aβ42/Aβ40 ratio was significantly decreased in AD and FTLD compared to cognitively normal participants, reflecting amyloid pathology. Elevated *p*‐tau181 levels in AD and FTLD patients indicate tau‐related changes, while higher NfL levels in FTLD compared to AD highlight its role in neurodegeneration. Ridge regression analyses identified biomarker‐specific cognitive associations: in AD, *p*‐tau181 and NfL correlated with memory, global cognition, and functionality impairments, whereas in FTLD, these markers primarily reflected global cognitive decline. Machine learning models trained with AT(N) biomarkers achieved high diagnostic accuracy (ROC‐AUC: AD=83%, FTLD=88%). Integrating neuroimaging further enhanced performance (AD=88%, FTLD=89%) and revealed structural and functional brain changes in temporoparietal and frontotemporal regions associated with biomarker alterations. These findings highlight the global utility of AT(N) biomarkers when integrated with clinical and neuroanatomical assessments for enhancing dementia diagnosis in diverse populations.

CDA is supported by ANID/FONDECYT Regular 1210622, ANID/PIA/ANILLOS ACT210096, Alzheimer's Association (AARGD‐24‐ 1310017), ANID/FOVI240065 and ANID/Proyecto Exploracion 13240170. Data in this manuscript were collected by the MULTI‐PARTNER CONSORTIUM TO EXPAND DEMENTIA RESEARCH IN LATIN AMERICA (ReDLat), supported by NIH research grant R01 AG057234 funded by the National Institute on Aging (NIA) and the Fogarty International Center (FIC), an Alzheimer's Association grant (SG‐20‐725707‐ReDLat), the Rainwater Charitable Foundation, and the Global Brain Health Institute with additional support from the Bluefield Project to Cure Frontotemporal Dementia, an NIH Contract (75N95022C00031), and NIA under award numbers R01 AG075775, R01 AG082056, and R01 AG083799. The content is solely the responsibility of the authors and does not represent the official views of the National Institutes of Health, Alzheimer's Association, Rainwater Charitable Foundation, Bluefield Project to Cure Frontotemporal Dementia or Global Brain Health Institute.

